# Patterns and Predictors of Repeated Medication Nonadherence in Glaucoma: A Population-Based Study

**DOI:** 10.3390/jcm15208038

**Published:** 2026-10-16

**Authors:** Tal Corina Sela, Yuval Kozlov, Noa Geffen, Amit Meshi, Guy Melamed, Amir Sternfeld, Yael Sharon, Alon Zahavi

**Affiliations:** 1 FOKUS AUGE Ophthalmology Clinic, 83022 Rosenheim, Germany; 2 Department of Military Medicine, Faculty of Medicine, The Hebrew University of Jerusalem, Jerusalem 9190500, Israel; uval.kozlov@mail.huji.ac.il; 3 Department of Ophthalmology, Rabin Medical Center–Beilinson Hospital, Petach Tikva 4941492, Israel; noatal1122@gmail.com (N.G.); amitmeshi@gmail.com (A.M.); shtery@gmail.com (A.S.); yaelpauker@gmail.com (Y.S.); 4 Gray Faculty of Medical and Health Sciences, Tel Aviv University, Tel Aviv 6997801, Israel; melamed_gu@mac.org.il; 5 Kahn Sagol Maccabi (KSM) Research & Innovation Center, Maccabi Healthcare Services, Tel Aviv 6812509, Israel; 6 Laboratory of Eye Research, Felsenstein Medical Research Center, Rabin Medical Center, Petach Tikva 4941492, Israel

**Keywords:** glaucoma, medication adherence, medication nonadherence, prescription refill, cohort studies

## Abstract

**Background/Objectives:** We aimed to evaluate nonadherence to topical glaucoma therapy and identify risk factors for repeated prescription fulfillment failure in a large national population-based cohort. **Methods:** The electronic medical records from a large health maintenance organization were retrospectively reviewed for all patients aged 18 years or older with a diagnosis of glaucoma from 2000 to 2023. Data on patients who had purchased at least one anti-glaucoma medication were retrieved. Compliance was defined as prescription fulfillment within predefined expiration windows of 1, 2, and 3 months. Repeated nonadherence was defined as three unfulfilled prescriptions within a 3-month expiration window. Multivariable Cox proportional hazards models were used to identify risk factors for repeated failure. **Results:** Among 40,200 treated patients, ≥80% cumulative prescription fulfillment was achieved in 80.3% of the population under a 3-month expiration definition. Under a strict 1-month definition, the incidence rate of the third compliance failure was 335 per 1000 person-years. In multivariable Cox analysis, obesity (HR 1.37, 95% CI 1.29–1.45) and current smoking (HR 1.07, 95% CI 1.04–1.11) were independently associated with increased risk of repeated failure. Older age (≥70 years: HR 0.73, 95% CI 0.62–0.87) and higher baseline intraocular pressure (≥25 mmHg: HR 0.65, 95% CI 0.61–0.68) were associated with reduced risk. Initiation with prostaglandin analogs was associated with lower hazard compared with alpha-2 adrenergic agonists (HR 0.53, 95% CI 0.49–0.57), whereas treatment alterations (≥3 changes) were associated with increased hazard (HR 1.64, 95% CI 1.39–1.95). **Conclusions:** Repeated nonadherence to topical glaucoma therapy is common and is associated with identifiable behavioral and treatment-related factors. Recognition of patients at elevated risk may support targeted strategies to enhance long-term treatment stability.

## 1. Introduction

Glaucoma is a leading cause of irreversible blindness worldwide [1]. It is characterized by progressive optic neuropathy that often remains asymptomatic until advanced stages. Reduction in intraocular pressure remains the only proven strategy to slow disease progression, and for most patients this is achieved through long-term topical therapy [2]. Because glaucoma is chronic and frequently asymptomatic in its early phases, sustained adherence to prescribed therapy is critical to preserving visual function [3].

Despite the importance of consistent treatment, nonadherence to topical glaucoma medications is common. Prior studies have reported substantial variability in adherence rates, influenced by factors such as age, socioeconomic status, medication burden, and disease severity [4,5,6,7,8]. However, many investigations have relied on small cohorts, short follow-up periods, or cross-sectional designs. Moreover, most studies evaluate overall adherence rates without examining repeated refill failures, which may better reflect clinically meaningful treatment instability [3,9,10]. Most prior investigations have assessed adherence using medication possession ratio, proportion of days covered, cross-sectional refill estimates, or isolated refill gaps. While informative, these approaches primarily capture aggregate medication coverage rather than patterns of sustained treatment interruption. Few large-scale population-based studies have modeled repeated refill failures longitudinally within the same cohort, directly compared alternative operational refill window definitions, or integrated behavioral factors with ophthalmic clinical variables over extended follow-up.

The present study aimed to evaluate adherence to topical anti-glaucoma therapy in a large national healthcare cohort and identify factors associated with repeated prescription fulfillment failure. By modeling repeated refill failures longitudinally within a large population-based dataset and directly comparing alternative operational refill window definitions, this analysis provides a more clinically relevant characterization of treatment instability than traditional aggregate adherence metrics. These findings help delineate patterns of sustained treatment interruption in glaucoma and highlight patient groups that may benefit from targeted adherence support strategies.

## 2. Materials and Methods

We conducted a retrospective population-based cohort study evaluating nonadherence to topical anti-glaucoma therapy using longitudinal electronic medical record data.

Data were obtained from Maccabi Health Services (MHS), the second largest health maintenance organization in Israel, providing comprehensive healthcare coverage to approximately 2.6 million members. The MHS database includes demographic information, diagnostic codes, prescription records, medication purchase data, laboratory results, outpatient visits, procedures, and hospitalizations.

All analyses were conducted within the secure MHS research environment using coded patient identifiers. The study period extended from 1 January 2000 to 31 December 2023. This study was approved by the Maccabi Health Services Institutional Review Board (approval number 0082-23-MHS). Given the retrospective design and use of de-identified electronic medical record data, the requirement for informed consent was waived. The study was conducted in accordance with the Declaration of Helsinki and applicable national data protection regulations.

Cohort identification and data extraction were performed electronically using predefined database queries within the MHS research environment. Adult patients aged 18 years or older with a diagnosis of glaucoma were identified using ICD-9 code 365 and corresponding internal MHS diagnostic codes. De-identified records meeting the predefined criteria were retrieved from the MHS database for subsequent processing and analysis. The index date was defined as the first recorded glaucoma diagnosis.

Patients were included if they had at least one documented purchase of an anti-glaucoma medication classified under Anatomical Therapeutic Chemical (ATC) level 5 codes S01E. Patients were excluded if they had less than one year of continuous membership in MHS prior to diagnosis or had less than one year of follow-up after diagnosis. Continuous membership was defined as uninterrupted enrollment in MHS for at least 365 consecutive days prior to the index diagnosis date. Patients with missing residential information were also excluded to reduce potential misclassification of medication nonadherence, as medication purchases made abroad are not captured in the MHS pharmacy database.

Patients were followed from the index date until death, departure from MHS, or 31 December 2023, whichever occurred first.

Prescription fulfillment was defined as a documented purchase of a prescribed anti-glaucoma medication within predefined expiration windows of 1, 2, or 3 months following prescription issuance. In the MHS system, prescriptions are issued by the treating physician to match the quantity and dosing frequency required for the patient’s specific regimen, including bilateral treatment when applicable. Prescriptions are not valid beyond 3 months from the date of issuance. Accordingly, the 3-month window represents the maximal allowable prescription validity period. No additional grace period beyond the predefined expiration window was permitted in the definition of prescription fulfillment. Because a shorter expiration window may misclassify patients who still have an adequate remaining medication supply as nonadherent, whereas failure to purchase medication within the full 3-month validity period provides stronger evidence of a genuine absence of available treatment, the 3-month window was considered the most specific and clinically reliable indicator of true nonadherence. Therefore, the 3-month expiration window was prespecified as the primary definition, with 1- and 2-month windows analyzed as sensitivity definitions. Compliance was calculated as the proportion of fulfilled prescriptions relative to the total number of prescriptions during follow-up and was expressed as a percentage per patient-year.

Compliance failure was defined as failure to purchase a prescribed medication within the specified expiration window. Repeated compliance failure was defined as three occurrences of prescription non-fulfillment within the 3-month prescription expiration window.

Time-to-event analyses evaluated time from index date to first, second, and third compliance failure. Person-years were used to account for differences in follow-up duration between patients and were calculated as the sum of individual follow-up times, expressed in years, providing the denominator for incidence rates of compliance failure. For descriptive purposes, incidence rate was additionally converted to an approximate one-year cumulative probability using the standard relation 1 − e^(−rate)^, assuming an approximately constant hazard [11].

Demographic variables included age at diagnosis, sex, socioeconomic status, residential region, and demographic sector.

Systemic variables included body mass index, smoking status (current, former, or never), number of chronic medications, and major comorbid conditions.

Ophthalmic variables included the intraocular pressure of the eye with the higher measurement at diagnosis, frequency of intraocular pressure measurements during follow-up, frequency of visual field testing and optical coherence tomography imaging, frequency of ophthalmologist and glaucoma specialist visits, and history of glaucoma-related procedures or cataract surgery.

The first prescribed anti-glaucoma medication class (ATC level 5) was recorded. This classification level identifies the specific chemical substance or product, allowing clear differentiation between distinct agents, including fixed-dose combinations, and preventing misclassification between different medications. Treatment alteration was defined as ≥1 change to a different ATC level 5 agent occurring more than 90 days after the prior prescription without re-prescription of the previous agent within 90 days, a buffer period intended to accommodate reasonable overlap in medication supply between sequential prescriptions. All covariates were treated as fixed variables in the regression models. Demographic and systemic characteristics were defined at baseline (index date). Monitoring frequencies were summarized as annualized measures over follow-up and were not modeled as time-varying exposures.

Baseline characteristics and compliance distributions were summarized using descriptive statistics. Linear regression models were used to evaluate associations between covariates and percentage of fulfilled prescriptions within the 3-month expiration window. Multivariable models were adjusted for demographic and systemic confounders. Regression coefficients with 95% confidence intervals (CIs) and *p*-values were reported.

The Cox proportional hazards model was constructed to evaluate time to third compliance failure within the 3-month expiration window. Multivariable models were adjusted for age, sex, socioeconomic status, and smoking status. Hazard ratios (HRs) with 95% CIs and *p*-values were reported. Cumulative incidence curves were generated for first and repeated compliance failure events under the predefined expiration windows. All analyses were performed using R statistical software, version 4.4.0 (R Foundation for Statistical Computing, Vienna, Austria). Two-sided *p*-values < 0.05 were considered statistically significant.

## 3. Results

### 3.1. Cohort Characteristics

**Figure 1:**
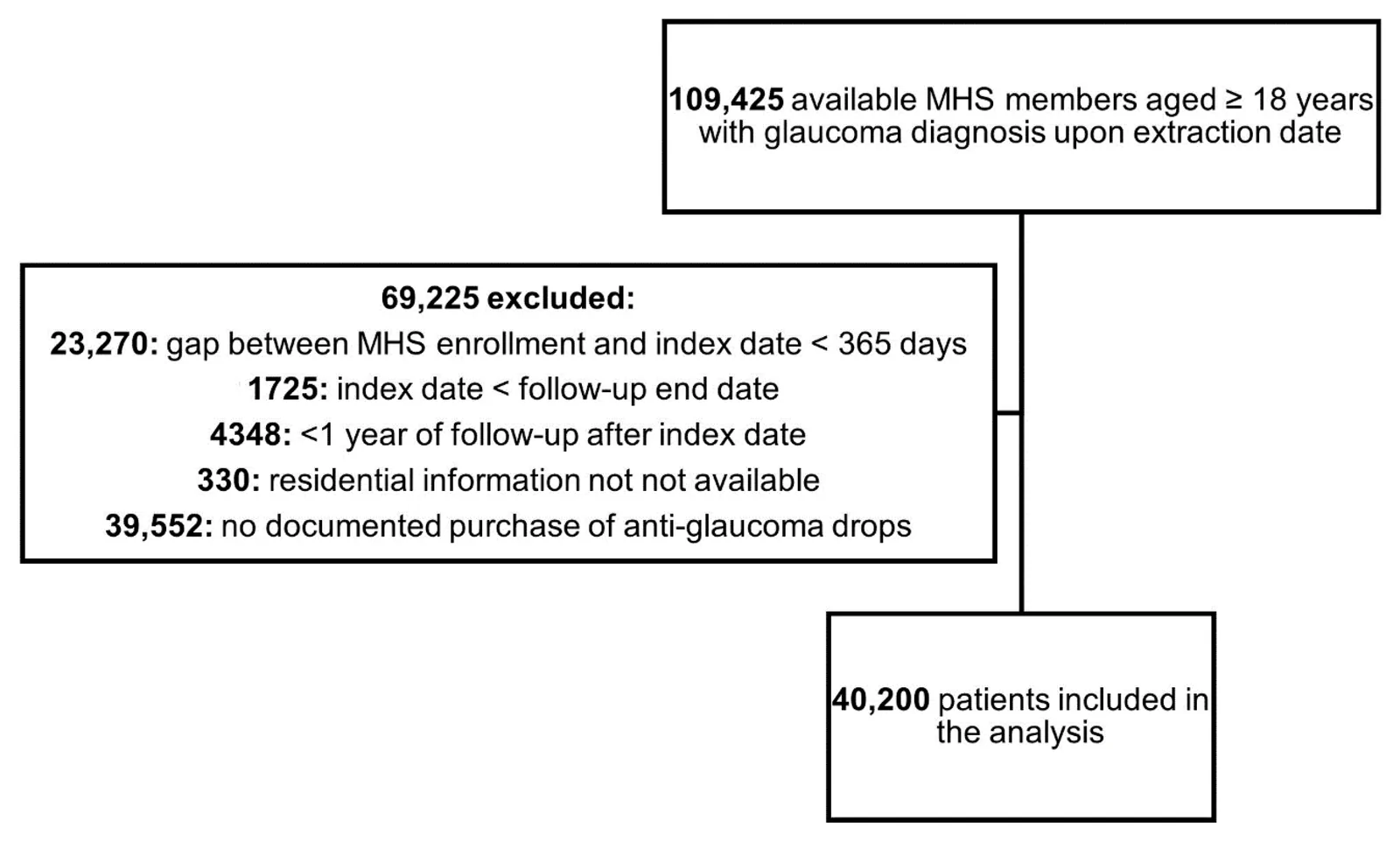
Flow diagram of cohort selection.

A total of 109,425 patients with a recorded diagnosis of glaucoma were identified during the study period. After applying eligibility criteria, 40,200 patients constituted the treated cohort included in the analysis. The cohort derivation process is shown in Figure 1. The baseline demographic and clinical characteristics of the treated cohort are presented in Table 1.

The majority of patients initiated therapy with prostaglandin analogs. At least one treatment alteration, defined as ≥1 change in ATC level 5 medication class, occurred in 15.8% of patients during follow-up. The analysis included 141,783 person-years of follow-up.

Of 109,425 patients with a recorded diagnosis of glaucoma, 40,200 met eligibility criteria and were included in the analysis (Figure 1).

### 3.2. Prescription Fulfillment and Compliance Distribution

Using the primary 3-month prescription expiration window, 80.3% of treated patients achieved ≥80% cumulative prescription fulfillment over follow-up. When applying a different operational definition of prescription expiration, a substantial variation in estimated adherence was observed: under 2-month and 1-month expiration definitions, ≥80% fulfillment was observed in 75.3% and 59.7% of patients, respectively. Table 2 presents the distribution of prescription fulfillment percentages across the predefined prescription expiration periods of 1, 2, and 3 months.

When examining intermediate adherence levels, 91.5%, 89.9%, and 84.5% of patients reached ≥60% cumulative prescription fulfillment using the 3-month, 2-month, and 1-month expiration definitions, respectively.

### 3.3. Predictors of Prescription Fulfillment

In multivariable linear regression analysis evaluating cumulative prescription fulfillment percentage within the 3-month expiration window, higher baseline intraocular pressure (IOP) demonstrated a graded association with improved compliance. Compared with IOP < 15 mmHg, a baseline IOP of 20–24 mmHg was associated with a 2.42% higher fulfillment rate (95% CI 1.86–2.98, *p* < 0.01), and IOP ≥ 25 mmHg was associated with a 3.93% higher fulfillment rate (95% CI 3.33–4.54, *p* < 0.01). Full regression coefficients are presented in Table 3.

Current smoking was associated with lower fulfillment rates (−1.30%, 95% CI −1.69 to −0.91, *p* < 0.01), and obesity was associated with lower compliance compared with normal BMI (−2.26%, 95% CI −2.84 to −1.69, *p* < 0.01).

Initiation with prostaglandin analogs or carbonic anhydrase inhibitors was associated with higher fulfillment compared with sympathomimetic agents (+1.75%, 95% CI 0.83–2.67, *p* < 0.01 and +1.11%, 95% CI 0.14–2.07, *p* = 0.02, respectively).

### 3.4. Incidence Rate of Compliance Failure

**Figure 2:**
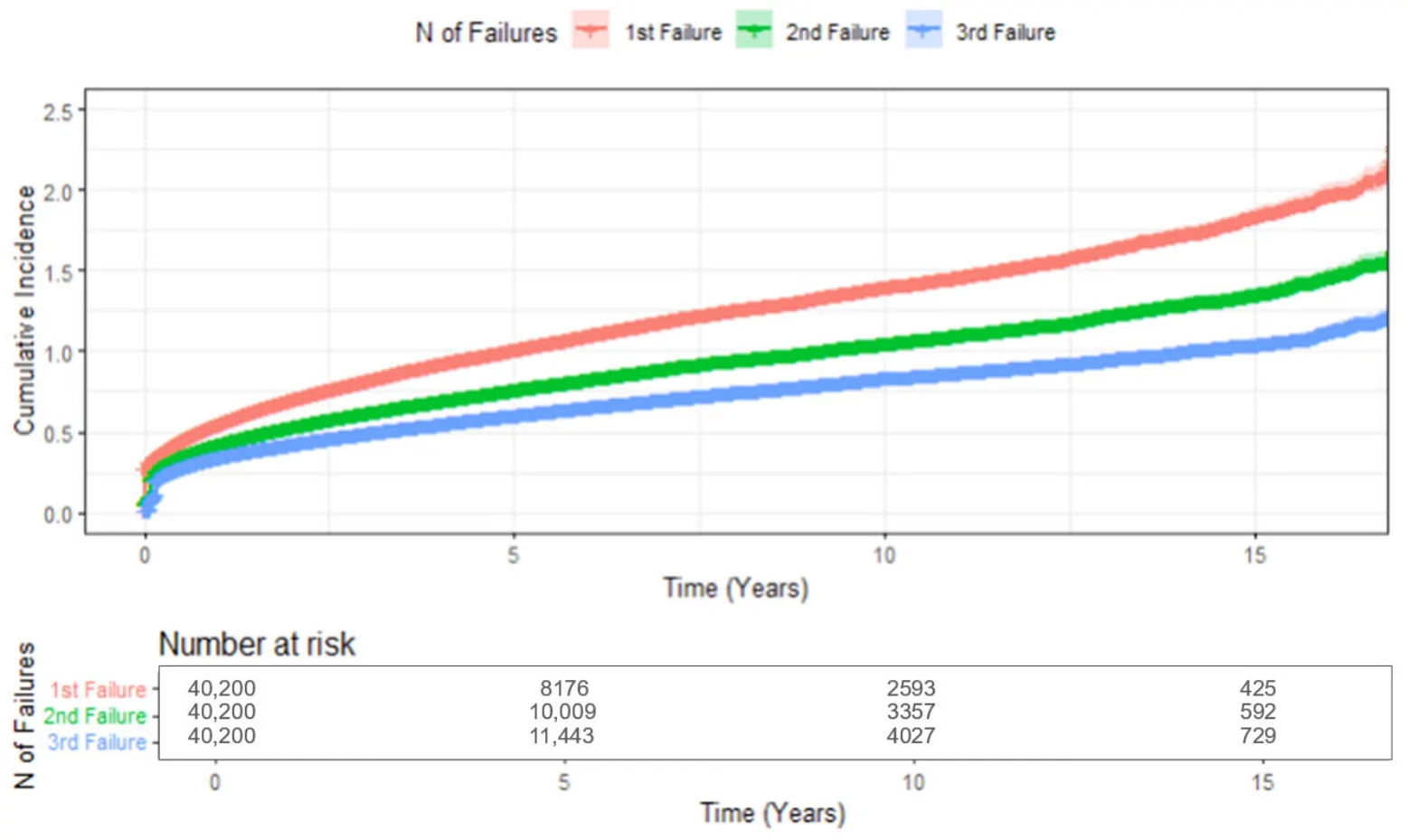
Cumulative incidence of one, two, and three compliance failure events under the 3-month prescription expiration definition. The curves represent time from index diagnosis to the occurrence of unfulfilled prescription events. Numbers at risk are shown below the x-axis.

Under the strict 1-month expiration definition, the incidence rate of first compliance failure was 818 per 1000 person-years, and the incidence rate of third compliance failure was 335 per 1000 person-years. For illustrative purposes, and assuming an approximately constant hazard, this corresponds to an approximate 28.5% probability of experiencing a third compliance failure within one year of follow-up [11]. Under the 3-month expiration definition, the compliance failure incidence rate per 1000 person-years was 237 for the first compliance failure and 122 for the third compliance failure. The cumulative incidence of one, two, or three compliance failure events under the 3-month expiration definition is shown in Figure 2 and demonstrates progressive accumulation over time.

### 3.5. Predictors of Repeated Compliance Failure

In multivariable Cox proportional hazards analysis evaluating time to third compliance failure under the 3-month expiration definition (Table 4), older age was associated with reduced risk. Compared with patients younger than 30 years, patients aged ≥70 years had a hazard ratio (HR) of 0.73 (95% CI 0.62–0.87, *p* < 0.01).

Obesity was associated with increased risk (HR 1.37, 95% CI 1.29–1.45, *p* < 0.01), and current smoking was associated with increased risk (HR 1.07, 95% CI 1.04–1.11, *p* < 0.01).

Higher baseline intraocular pressure was associated with a lower hazard of repeated failure. Compared with IOP < 15 mmHg, IOP ≥ 25 mmHg was associated with HR 0.65 (95% CI 0.61–0.68, *p* < 0.01).

Initiation with prostaglandin analogs was associated with a lower hazard of repeated failure compared with alpha-2 adrenergic agonists (HR 0.53, 95% CI 0.49–0.57, *p* < 0.01). Similar results were observed for carbonic anhydrase inhibitors and beta-blockers (HR 0.84, 95% CI 0.78–0.91, *p* < 0.01, and HR 0.85, 95% CI 0.79–0.92, *p* < 0.01, respectively). Treatment alterations demonstrated a dose–response association with increased hazard of repeated failure (≥3 alterations: HR 1.64, 95% CI 1.39–1.95, *p* < 0.01).

The Cox model included 17,325 events of a third compliance failure.

## 4. Discussion

In this large national population-based cohort of treated glaucoma patients, repeated prescription nonfulfillment was common and accumulated progressively over time, despite generally high levels of cumulative prescription fulfillment. Under the strict 1-month expiration definition, the incidence rate of third compliance failure reached 335 per 1000 person-years, indicating that recurrent treatment interruption affects a substantial subset of patients. Importantly, adherence estimates varied considerably according to the prescription expiration window applied, with broader refill definitions producing substantially higher apparent adherence rates. These findings suggest that conventional aggregate fulfillment measures may underestimate clinically meaningful treatment instability by failing to distinguish patients with sustained refill behavior from those experiencing recurrent interruptions. This distinction may identify a subgroup of patients whose adherence appears acceptable using conventional cumulative metrics yet is characterized by recurrent treatment interruption.

Prior studies evaluating adherence to topical glaucoma therapy have reported wide variability, often ranging from 40% to 80%, largely depending on methodology and duration of follow-up [6,9,12]. Many investigations have relied on medication possession ratio, proportion of days covered, self-reported adherence, or cross-sectional refill assessments [3,6,12]. While informative, these approaches primarily quantify overall medication coverage rather than patterns of recurrent treatment interruption. Large administrative database studies have demonstrated declining persistence over time, yet most have focused on isolated refill gaps or overall adherence metrics. In contrast, the present study focused on repeated prescription nonfulfillment events, which may better capture persistent treatment instability than aggregate adherence measures alone. By evaluating the third repeated failures within a large longitudinal cohort and directly comparing alternative refill-window definitions, this study identifies a subgroup of patients whose adherence may appear acceptable using conventional cumulative measures yet is characterized by recurrent treatment interruption.

Nonadherence was not uniformly distributed across the cohort but concentrated in identifiable subgroups. Importantly, the identified risk factors demonstrated consistent associations across both the time-to-event and cumulative fulfillment analyses, supporting the robustness and internal validity of the findings.

Younger age was independently associated with increased risk of repeated failure, consistent with prior reports linking younger patients to reduced refill persistence in glaucoma and other chronic diseases [6,13,14]. Younger individuals may perceive lower immediate risk from an often asymptomatic condition or may face competing occupational and social demands that interfere with sustained medication use. In contrast, older patients demonstrated a lower hazard of repeated failure, potentially reflecting greater health system engagement or heightened perception of disease severity.

Systemic health behaviors also emerged as relevant correlates. Obesity and current smoking were independently associated with increased risk of repeated nonadherence. Similar associations have been observed across multiple chronic medical conditions, suggesting that glaucoma adherence behavior may be embedded within broader behavioral and lifestyle patterns [7,15,16]. Recognition of these systemic factors may assist clinicians in identifying patients who could benefit from intensified adherence counseling or closer follow-up.

Higher baseline IOP was associated with a lower hazard of repeated failure. Patients presenting with more pronounced pressure elevation may perceive greater disease severity or receive more intensive counseling at diagnosis. However, disease severity itself may confound this association. Patients with higher IOP or more advanced disease are likely to undergo more frequent monitoring and clinical engagement, both of which may reinforce adherence behavior. Although prior literature has shown mixed findings regarding the relationship between disease severity and persistence, some studies suggest that patients with greater perceived visual risk demonstrate improved adherence [3].

The apparently protective associations observed for multiple comorbidities in both the linear regression and Cox analyses should be interpreted cautiously. These associations, as well as the contrasting directions observed for monitoring frequency (IOP, OCT, visual field testing) versus clinical visit frequency (ophthalmologist and glaucoma specialist visits), could reflect differences in healthcare utilization and disease surveillance rather than direct effects on adherence. Treatment alterations demonstrated a dose–response association with increased hazard of repeated failure. Frequent medication changes may increase regimen complexity, reduce patient confidence in treatment, or reflect intolerance or unstable disease. Polypharmacy and multi-drop regimens have previously been associated with reduced adherence in glaucoma [3,9,12]. Patients requiring repeated therapeutic adjustments may therefore represent a subgroup at particularly high risk for sustained nonadherence and may benefit from structured adherence support or regimen simplification when clinically feasible.

From a clinical perspective, these findings suggest that high overall adherence rates may mask meaningful instability in a subset of patients. While most treated patients achieved high fulfillment percentages under broader definitions, stricter criteria revealed a substantial burden of recurrent treatment interruption. Identification of patients at elevated risk, including younger individuals, smokers, those with obesity, and those undergoing frequent medication changes, may permit targeted strategies to enhance long-term therapeutic stability. In practice, this requires clinicians, including family physicians, who may encounter patients more frequently, to proactively identify individuals with nonadherence and discuss the underlying reasons with them. Where adherence to topical therapy remains persistently low, this may support earlier consideration of alternative treatment modalities, such as selective laser trabeculoplasty or surgery, to secure stable long-term IOP control. Given the chronic and frequently asymptomatic nature of glaucoma, repeated treatment gaps may contribute to cumulative disease progression, although this study did not directly assess visual outcomes.

This study has several strengths. To our knowledge, few prior studies have modeled repeated refill failures at a national scale with extended longitudinal follow-up. It leverages a large national health service database with comprehensive longitudinal prescription and purchase records over more than two decades. The use of objective pharmacy purchase data reduces recall bias and enhances measurement reliability compared with self-reported adherence studies. The population-based design enhances generalizability within similar healthcare systems. Modeling repeated failures provides a clinically meaningful measure of sustained nonadherence beyond isolated refill gaps. Inclusion of demographic, behavioral, systemic, and treatment-related variables allowed multivariable evaluation of factors associated with treatment instability.

Several limitations warrant consideration. Prescription fulfillment was used as a proxy for medication adherence and does not confirm actual drop instillation or correct administration technique. In addition, ophthalmic monitoring and procedural variables were derived from routinely collected administrative records and may not fully capture all testing, visits, or procedures performed outside coded healthcare pathways. Therefore, associations involving these variables should be interpreted cautiously. The analysis was restricted to patients who purchased at least one anti-glaucoma medication; therefore, primary non-initiation of therapy was not assessed. The definition of repeated nonadherence did not require the three qualifying non-fulfillment episodes to be consecutive, and the time interval between them was not directly evaluated. Three failures occurring in close succession may carry different clinical significance than failures spread across a longer period of follow-up. Although multivariable models adjusted for key demographic and clinical factors, residual confounding by disease severity, healthcare utilization patterns, or unmeasured psychosocial determinants may have influenced observed associations. Treatment alterations may reflect underlying disease complexity rather than causal determinants of adherence, and reverse causation cannot be excluded. Additionally, covariates were treated as fixed variables and were not modeled as time-varying exposures, which may limit the ability to capture dynamic changes in clinical status over follow-up. In particular, treatment alterations, monitoring frequency, and glaucoma-related procedures (cataract surgery, laser treatment, and trabeculectomy) were derived from events occurring throughout follow-up rather than strictly at baseline and may themselves be influenced by prior nonadherence. Our data did not allow reliable reconstruction of the precise temporal sequence between these clinical events and each qualifying non-fulfillment episode, precluding a formal time-varying covariate analysis. Associations involving these variables should therefore be interpreted with caution, as they may reflect consequences of nonadherence rather than risk factors for it. Moreover, in the Cox analyses, follow-up time was calculated from the index date (first glaucoma diagnosis) rather than from treatment initiation. Patients with a delay between diagnosis and their first prescription would accrue at-risk time before becoming eligible for a compliance failure event, which may underestimate the true incidence rate of compliance failure. This concern does not apply to the compliance-rate (linear regression) analysis, which was based on the percentage of prescription fulfillment across the treatment period. The proportional hazards assumption underlying the Cox models was not formally tested. Given the extended study period, hazard ratios may not have remained constant over the duration of follow-up. Finally, this observational design precludes causal inference and did not directly evaluate the relationship between repeated nonadherence and visual outcomes.

## 5. Conclusions

In this large national population-based cohort, repeated nonadherence to topical glaucoma therapy was frequent despite high overall prescription fulfillment rates under broader definitions. This finding at a national level underscores the importance of clinicians identifying glaucoma patients with low adherence, engaging them in conversation about this issue, and considering therapy adjustment to optimize IOP control. Recurrent treatment interruption was concentrated in identifiable subgroups, including younger patients, individuals with obesity, current smokers, and those undergoing multiple medication changes. Identification of patients at elevated risk of prescription non-fulfillment may facilitate targeted adherence support and help inform strategies to promote long-term treatment stability in glaucoma care.

## Figures and Tables

**Table 1:** Baseline demographics and clinical characteristics of the study population.

Variable		Total Cohort = 40,200 *n* (%)
Age (years)	<30	380 (0.95%)
	30–39	1104 (2.75%)
	40–49	4058 (10.1%)
	50–59	8322 (20.7%)
	60–69	12,939 (32.2%)
	≥70	13,397 (33.3%)
Gender	Female	20,355 (50.6%)
	Male	19,845 (49.4%)
Follow-up duration (years)	<5	7967 (19.8%)
	5–9	9854 (24.5%)
	10–14	8282 (20.6%)
	15–19	7037 (17.5%)
	≥20	7060 (17.6%)
MHS membership duration (years)	<5	5099 (12.7%)
	5–9	5568 (13.8%)
	10–14	5713 (14.2%)
	15–19	5602 (13.9%)
	≥20	18,218 (45.3%)
Reason for end of follow-up	Follow-up until 31 December 2023.	31,234 (77.7%)
	Death	8343 (20.8%)
	Leaving MHS	623 (1.55%)
Socioeconomic status (1–10)	Low (1–3)	5396 (13.4%)
	Middle (4–6)	14,058 (35.0%)
	High (7–10)	20,157 (50.1%)
Demographic sector	Other	30,904 (76.9%)
	Orthodox	2032 (5.05%)
	Arab	1189 (2.96%)
	Russian	5105 (12.7%)
	National-Jewish	970 (2.41%)
Residential region	Central	37,068 (92.2%)
	Peripheral	3132 (7.79%)
BMI (kg/m^2^)	Underweight (<18.5)	197 (0.49%)
	Normal weight (18.5–24.9)	6694 (16.6%)
	Overweight (25.0–29.9)	10,719 (26.7%)
	(Obese ≥30)	8576 (21.3%)
Smoking status	Non-smokers	22,610 (56.2%)
	Previous smokers	3886 (9.67%)
	Current smokers	13,704 (34.1%)
Systemic comorbidities	Cardiovascular	17,773 (44.2%)
	Endocrine	15,824 (39.4%)
	Connective tissue	7784 (19.4%)
	Gastrointestinal	7395 (18.4%)
	Hematologic	1989 (4.95%)
	Psychiatric	5956 (14.8%)
	Genitourinary	5605 (13.9%)
	Neoplasms	5581 (13.9%)
	Neurologic	10,257 (25.5%)
	Respiratory	2989 (7.44%)
	Pregnancy-related	160 (0.4%)
	Cutaneous	1097 (2.73%)
IOP at diagnosis (mmHg)	Not available	2601 (6.47%)
	<15	14,627 (36.4%)
	15–19	8694 (21.6%)
	20–24	7580 (18.9%)
	≥25	6428 (16.0%)

Percentages were calculated using the total cohort (*n* = 40,200) as the denominator. In cases of missing data, percentages may not sum to 100%. BMI, body mass index; IOP, intraocular pressure; MHS, Maccabi Health Services.

**Table 2:** Distribution of prescription fulfillment percentages according to predefined prescription expiration periods.

Predefined Prescription Expiration Period	Percentage of Prescription Fulfillment (%)	Total Cohort = 40,200 *n* (%)
1 month	0–19	773 (1.92%)
	20–39	1625 (4.04%)
	40–59	3839 (9.55%)
	60–79	9966 (24.8%)
	80–100	23,997 (59.7%)
2 months	0–19	592 (1.47%)
	20–39	1177 (2.93%)
	40–59	2280 (5.67%)
	60–79	5891 (14.6%)
	80–100	30,260 (75.3%)
3 months	0–19	529 (1.32%)
	20–39	1016 (2.53%)
	40–59	1862 (4.63%)
	60–79	4506 (11.2%)
	80–100	32,287 (80.3%)

**Table 3:** Multivariable linear regression analysis of factors associated with the percentage of fulfilled prescriptions within the 3-month prescription expiration window.

Variable		Estimate	95% Confidence Interval		*p* Value
Age (years)	<30	0	Reference		
	30–39	−1.38	−3.54	0.79	0.21
	40–49	−0.10	−2.05	1.85	0.92
	50–59	0.50	−1.40	2.40	0.61
	60–69	1.20	−0.69	3.09	0.21
	≥70	1.73	−0.16	3.61	0.07
Gender	Female	0	Reference		
	Male	0.17	−0.20	0.53	0.37
BMI (kg/m^2^)	Normal weight (18.5–24.9)	0	Reference		
	Underweight (<18.5)	0.06	−2.49	2.60	0.97
	Overweight (25.0–29.9)	−0.97	−1.52	−0.42	0.00
	Obese (≥30)	−2.26	−2.84	−1.69	0.00
Socioeconomic status (1–10)	Low (1–3)	0	Reference		
	Middle (4–6)	−0.45	−1.03	0.13	0.13
	High (7–10)	0.66	0.10	1.21	0.02
Demographic sector	Other	0	Reference		
	Orthodox	0.42	−0.42	1.25	0.33
	Arab	−0.35	−1.42	0.73	0.53
	Russian	−0.59	−1.14	−0.04	0.03
	National-Jewish	0.30	−0.88	1.49	0.61
Residential region	Central	0	Reference		
	Peripheral	1.05	0.38	1.73	0.00
Smoking status	Non-smoker	0	Reference		
	Current smoker	−1.30	−1.69	−0.91	0.00
	Previous smokers	0.49	−0.14	1.12	0.13
Comorbidities	No diagnosis	0	Reference		
	Cardiovascular	1.84	1.48	2.21	0.00
	Endocrine	1.63	1.26	2.01	0.00
	Connective tissue	2.20	1.74	2.66	0.00
	Gastrointestinal	1.84	1.37	2.31	0.00
	Hematologic	1.04	0.21	1.88	0.01
	Psychiatric	0.78	0.27	1.29	0.00
	Genitourinary	1.55	1.03	2.08	0.00
	Neoplasms	2.73	2.21	3.25	0.00
	Neurologic	2.57	2.16	2.99	0.00
	Respiratory	1.58	0.89	2.27	0.00
	Pregnancy-related	2.48	−0.40	5.36	0.09
	Cutaneous	0.92	−0.19	2.03	0.11
Children with glaucoma	No	0	Reference		
	Yes	5.19	0.54	9.85	0.03
Pregnancy (*n* of conceptions after index date)	0	0	Reference		
	1	−0.15	−2.77	2.48	0.91
	2	−1.97	−5.78	1.85	0.31
	3	−8.86	−14.60	−3.11	0.00
	4	−5.91	−15.29	3.47	0.22
	5+	−0.15	−8.27	7.98	0.97
IOP at diagnosis (mmHg)	<15	0	Reference		
	15–19	0.93	0.43	1.44	0.00
	20–24	2.42	1.86	2.98	0.00
	≥25	3.93	3.33	4.54	0.00
IOP measurement frequency (*n* per year)	<2	0	Reference		
	2–4	5.21	4.81	5.62	0.00
	≥5	10.20	9.70	10.70	0.00
OCT frequency (*n* per year)	0	0	Reference		
	1	3.16	1.74	4.58	0.00
	2	3.58	−0.23	7.39	0.07
	≥3	6.38	−1.74	14.50	0.12
Visual field frequency (*n* per year)	0	0	Reference		
	1	5.46	3.60	7.33	0.00
	≥2	9.18	2.95	15.41	0.00
Ophthalmologist visit frequency (*n* per year)	0	0	Reference		
	1	0.58	−0.01	1.17	0.06
	2	−1.19	−2.73	0.34	0.13
	≥3	−1.78	−3.98	0.41	0.11
Glaucoma specialist visit frequency (*n* per year)	0	0	Reference		
	1	−0.03	−1.77	1.71	0.97
	≥2	−0.05	−4.19	4.09	0.98
Ophthalmology admissions (*n* per year)	0	0	Reference		
	1	−0.63	−7.06	5.79	0.85
Phacoemulsification before index date	No	0	Reference		
	Yes	2.43	−3.99	8.86	0.46
Phacoemulsification after index date	No	0	Reference		
	Yes	−2.44	−4.15	−0.74	0.01
Laser therapy * after index date	No	0	Reference		
	Yes	2.57	−0.79	5.93	0.13
Trabeculectomy after index date	No	0	Reference		
	Yes	3.23	−0.31	6.76	0.07
First medication	Sympathomimetic	0	Reference		
	Parasympathomimetic	−1.54	−3.43	0.34	0.11
	Carbonic anhydrase inhibitor	1.11	0.14	2.07	0.02
	Beta blocker	−0.42	−1.36	0.51	0.38
	Prostaglandin analogue	1.75	0.83	2.67	0.00
Treatment alterations (*n*)	0	0	Reference		
	1	1.91	1.33	2.49	0.00
	2	4.46	3.55	5.36	0.00
	≥3	2.80	0.38	5.22	0.02

Regression coefficients represent the absolute change in fulfillment percentage relative to the reference category for each variable. Regression coefficients that reached statistical significance are highlighted in yellow. Positive estimates indicate higher fulfillment, and negative estimates indicate lower fulfillment (highlighted in green and red if reaching statistical significance, respectively). The 3-month expiration window was prespecified as the primary compliance definition. * Laser therapy includes laser iridotomy and selective laser trabeculoplasty (SLT). BMI, body mass index; IOP, intraocular pressure; OCT, optical coherence tomography.

**Table 4:** Multivariable Cox regression analysis of risk factors for three repeated failures to fulfill prescriptions within the 3-month prescription expiration window.

Variable		HR	95% Confidence Interval		*p* Value
Age (years)	<30	1.00	Reference		
	30–39	0.93	0.77	1.12	0.43
	40–49	0.90	0.76	1.06	0.21
	50–59	0.93	0.79	1.09	0.37
	60–69	0.88	0.75	1.04	0.14
	≥70	0.73	0.62	0.87	0.00
Gender	Female	1.00	Reference		
	Male	0.97	0.94	1.00	0.04
BMI (kg/m^2^)	Normal weight (18.5–24.9)	1.00	Reference		
	Underweight (<18.5)	1.19	0.91	1.57	0.21
	Overweight (25.0–29.9)	1.13	1.06	1.20	0.00
	Obese (≥30)	1.37	1.29	1.45	0.00
Socioeconomic status (1–10)	Low (1–3)	1.00	Reference		
	Middle (4–6)	0.95	0.89	1.01	0.12
	High (7–10)	0.84	0.78	0.90	0.00
Demographic sector	Other	1.00	Reference		
	Orthodox	1.06	0.99	1.14	0.08
	Arab	0.95	0.87	1.04	0.25
	Russian	1.09	1.04	1.14	0.00
	National-Jewish	0.99	0.90	1.09	0.81
Residential region	Central	1.00	Reference		
	Peripheral	0.90	0.85	0.96	0.00
Smoking status	Non-smoker	1.00	Reference		
	Current smoker	1.07	1.04	1.11	0.00
	Previous smokers	0.75	0.71	0.79	0.00
Comorbidities	No diagnosis	1.00	Reference		
	Cardiovascular	0.56	0.54	0.58	0.00
	Endocrine	0.55	0.54	0.57	0.00
	Connective tissue	0.55	0.53	0.58	0.00
	Gastrointestinal	0.58	0.55	0.60	0.00
	Hematologic	0.55	0.50	0.60	0.00
	Psychiatric	0.61	0.58	0.64	0.00
	Genitourinary	0.60	0.58	0.64	0.00
	Neoplasms	0.53	0.50	0.56	0.00
	Neurologic	0.51	0.49	0.53	0.00
	Respiratory	0.66	0.62	0.71	0.00
	Pregnancy-related	0.62	0.46	0.83	0.00
	Cutaneous	0.63	0.56	0.70	0.00
Children with glaucoma	No	1.00	Reference		
	Yes	0.70	0.44	1.12	0.14
Pregnancy (*n* of conceptions after index date)	0	1.00	Reference		
	1	0.97	0.77	1.23	0.81
	2	1.30	0.95	1.77	0.10
	3	1.24	0.74	2.05	0.41
	4	2.07	1.15	3.74	0.02
	5+	1.18	0.56	2.48	0.66
IOP at diagnosis (mmHg)	<15	1.00	Reference		
	15–19	0.98	0.95	1.02	0.42
	20–24	0.72	0.69	0.75	0.00
	≥25	0.65	0.61	0.68	0.00
IOP measurement frequency (*n* per year)	<2	1.00	Reference		
	2–4	0.80	0.78	0.83	0.00
	≥5	0.44	0.42	0.46	0.00
OCT frequency (*n* per year)	0	1.00	Reference		
	1	0.47	0.40	0.55	0.00
	2	0.31	0.18	0.52	0.00
	≥3	0.41	0.16	1.10	0.08
Visual field frequency (*n* per year)	0	1.00	Reference		
	1	0.30	0.23	0.39	0.00
	≥2	0.07	0.01	0.52	0.01
Ophthalmologist visit frequency (*n* per year)	0	1.00	Reference		
	1	1.34	1.29	1.40	0.00
	2	1.59	1.43	1.77	0.00
	≥3	1.60	1.38	1.86	0.00
Glaucoma specialist visit frequency (*n* per year)	0	1.00	Reference		
	1	1.32	1.16	1.51	0.00
	≥2	2.08	1.58	2.75	0.00
Ophthalmology admissions (*n* per year)	0	1.00	Reference		
	1	0.82	0.46	1.49	0.52
Phacoemulsification before index date	No	1.00	Reference		
	Yes	1.13	0.68	1.87	0.65
Phacoemulsification after index date	No	1.00	Reference		
	Yes	1.86	1.66	2.07	0.00
Laser therapy * after index date	No	1.00	Reference		
	Yes	1.42	1.13	1.78	0.00
Trabeculectomy after index date	No	1.00	Reference		
	Yes	1.70	1.36	2.13	0.00
First medication	Sympathomimetic	1.00	Reference		
	Parasympathomimetic	1.14	0.97	1.34	0.10
	Carbonic anhydrase inhibitor	0.84	0.78	0.91	0.00
	Beta blocker	0.85	0.79	0.92	0.00
	Prostaglandin analogue	0.53	0.49	0.57	0.00
Treatment alterations (*n*)	0	1.00	Reference		
	1	1.26	1.20	1.31	0.00
	2	1.42	1.33	1.52	0.00
	≥3	1.64	1.39	1.95	0.00

Regression coefficients represent the hazard ratio (HR) for repeated failures relative to the reference category for each variable. Regression coefficients that reached statistical significance are highlighted in yellow; HR < 1 indicates a lower risk of adherence failure, and HR > 1 indicates a higher risk (highlighted in green and red if reaching statistical significance, respectively). * Laser therapy includes laser iridotomy and selective laser trabeculoplasty (SLT). BMI, body mass index; HR, hazard ratio; IOP, intraocular pressure; OCT, optical coherence tomography.

## Data Availability

The individual-level data supporting the findings of this study are not publicly available owing to patient privacy and institutional data-protection restrictions. Access to the de-identified data may be considered upon reasonable request, subject to approval by Maccabi Healthcare Services and the relevant ethics and data-governance authorities.
